# The atherogenic index of plasma is associated with subclinical cardiac systolic dysfunction among obese patients: a cross-sectional study

**DOI:** 10.3389/fnut.2026.1845459

**Published:** 2026-06-29

**Authors:** Guang-an Li, Jun Huang, Yan-jiao Wang

**Affiliations:** 1 Department of Cardiovascular Ultrasound, The Third Affiliated Hospital of Nanjing Medical University, Changzhou, China; 2 Department of Ultrasound, Peking Union Medical College Hospital, Beijing, China

**Keywords:** atherogenic index of plasma, global longitudinal strain, lipid-related biomarker, obesity, subclinical cardiac systolic dysfunction

## Abstract

**Background:**

Atherogenic index of plasma (AIP) is a novel and sensitive lipid-related biomarker closely associated with metabolic disorders and cardiovascular risk. Obesity is an independent risk factor for subclinical cardiac systolic dysfunction (SD), while the potential association between AIP and early cardiac systolic impairment in obese populations remains unclear. This study aimed to investigate the relationship between AIP and subclinical cardiac SD in obese adults.

**Methods:**

A cross-sectional study was conducted among 1,070 obese patients between 2019 and 2024. Clinical and biochemical parameters of obese patients were collected on the same day as echocardiographic image acquisition. Subclinical cardiac SD was defined via left ventricular (LV) global longitudinal strain (GLS) from speckle-tracking echocardiography. The logarithm of triglycerides/high-density lipoprotein cholesterol ratio is AIP. Patients were stratified by AIP levels, and regression models were constructed to explore the association between the AIP and subclinical cardiac SD in obese patients.

**Results:**

After patients were stratified by AIP levels AIP quartiles, the LV GLS absolute value in obese individuals with high AIP levels was significantly reduced compared to obese individuals with low AIP levels (*p* < 0.001). After the adjustment for the effects of body mass index, heart rate, sex, LV hypertrophy, LV ejection fraction, age, presence of hypertension, presence of diabetes, blood urea nitrogen and serum creatinine level in the multivariate regression model, AIP > 0.3661 was an independent influencing factor for absolute LV GLS < 20%.

**Conclusion:**

Elevated AIP is independently associated with subclinical cardiac SD among obese individuals. AIP may serve as a convenient and trustworthy biomarker for early screening of subclinical cardiac SD among obese individuals, which contributes to the early identification and prevention of obesity-related cardiac impairment.

## Introduction

Obesity, as a metabolic disease, manifests because of the interaction between the body’s genetic susceptibility to weight gain and environmental influences (1). In recent years, its global prevalence has gradually increased. Moreover, it has become a major driving factor for the increase in chronic noncommunicable diseases worldwide (2) and imposes an enormous burden on global health care systems (3). Obesity is related to the occurrence and pathogenesis of many chronic disorders (e.g., diabetes mellitus, cardiovascular disease, obstructive sleep apnoea syndrome, dyslipidaemia, and nonalcoholic fatty liver disorder) (4–7). Obesity can result in a reduction in left ventricular (LV) systolic function (8). Moreover, obesity has a negative influence on LV diastolic function (9). Currently, studies have shown that LV systolic function among obese individuals is still impaired in the subclinical stage with preserved LV ejection fraction (LVEF), which is referred to as subclinical LV systolic dysfunction (SD) (10, 11).

The logarithm of triglycerides (TG)/high-density lipoprotein cholesterol (HDL-C) ratio is AhIP, which is an innovative indicator for evaluating the occurrence of atherosclerosis and cardiovascular disease (12, 13). Investigations illustrated that the AIP has a nonlinear relationship with the incidence of hypertension (HT) and is a significant indicator of HT, which is particularly significant in the population with 45–60 years old (14). Additionally, existing studies have shown that after excluding the interference of confounding factors, the AIP is significantly connected to the prediabetes risk (15) and significantly linked to the incidence of type 2 diabetes (T2D) (16). Nevertheless, no investigations have examined whether the AIP can identify the occurrence of subclinical LV SD among obese patients. Thus, this investigation used speckle-tracking echocardiography (STE) to estimate LV systolic function among obese patients, explored the link between AIP and subclinical LV SD among obese patients, and assessed its reference value in identifying subclinical LV SD among obese patients.

## Methods

### Study population

**Figure 1:**
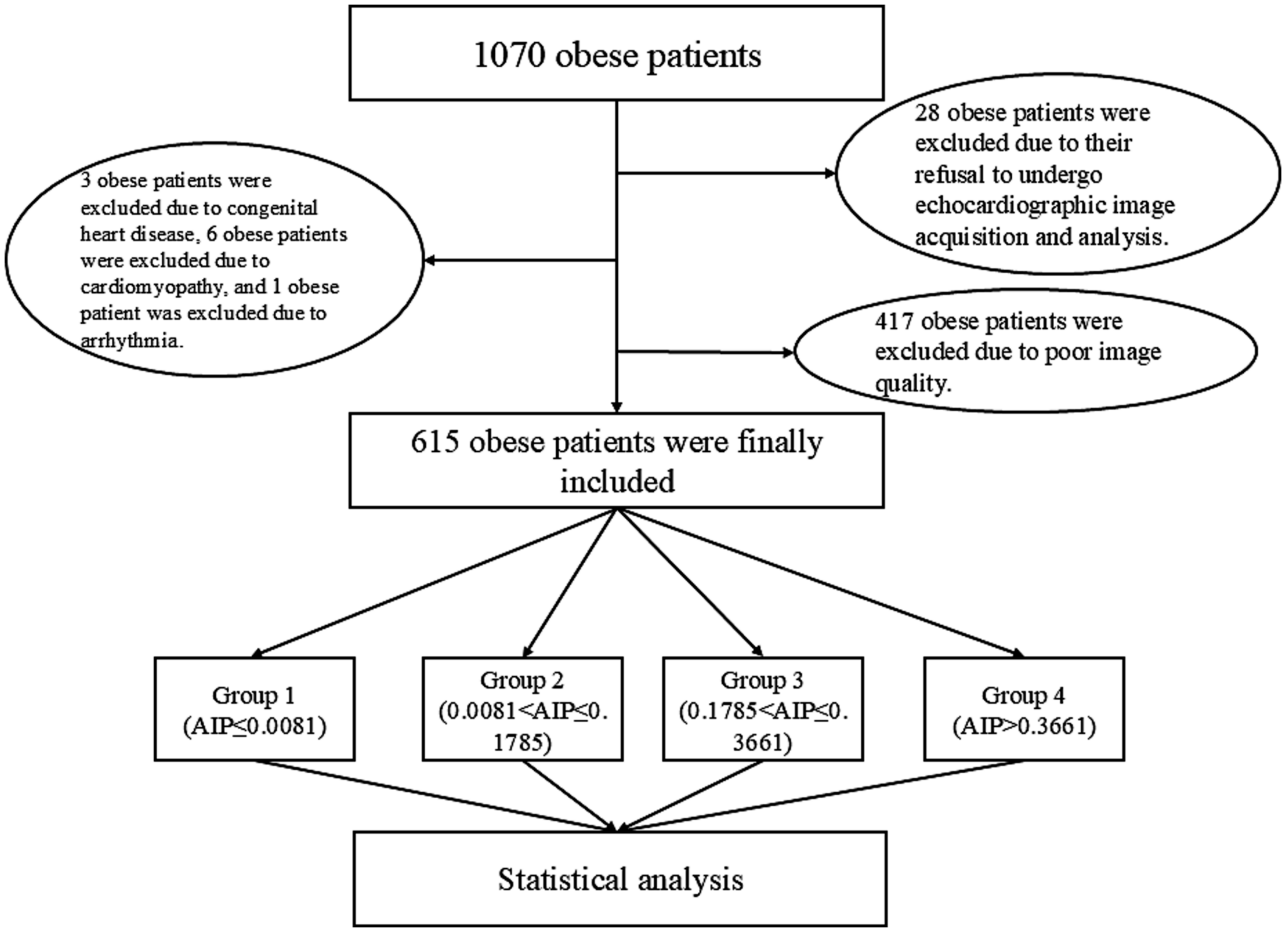
Population inclusion and exclusion of the study cohort.

Prior to the initiation of the present study, 1,070 obese patients were enrolled. All participants were obese patients hospitalized in the Bariatric and Metabolic Division of Weight Management Center, the Third Affiliated Hospital of Nanjing Medical University, between 2019 and 2024. The diagnostic criterion for obese patients is a BMI > 30 kg/m^2^ (17). Medical histories were collected prior to study initiation. Obese patients with a history of cardiomyopathy, congenital heart disease, arrhythmia, valvular heart disease, thyroid diseases or neoplastic diseases were excluded from image acquisition. Individuals newly diagnosed with coronary heart disease, cardiomyopathy or arrhythmia during imaging examination were also excluded. Some participants were excluded for refusing to undergo echocardiographic image collection. In addition, obese patients with unqualified ultrasound images that could not be analyzed owing to excessive subcutaneous fat were further excluded (Figure 1).

The ethics committee of the Third Affiliated Hospital of Nanjing Medical University gave its approval to this investigation. All obese patients were informed and consulted prior to image acquisition, and informed consent was obtained.

### Clinical and biochemical parameters

All obese patients provided their demographic information, such as sex, age, height, and weight, on the day of their echocardiographic examination and their body mass index (BMI) and body surface area (BSA) were examined depending on this information. All obese patients had their systolic (SBP) and diastolic (DBP) blood pressure evaluated before echocardiographic examination. The presence of complications such as diabetes, HT or dyslipidaemia in each obese patient was recorded. Additionally, collecting the fasting blood samples was conducted in the morning of the same day and submitted to the clinical laboratory to analyze the biochemical parameters, such as HDL-C, TG, total cholesterol (TC), low-density lipoprotein cholesterol (LDL-C), fasting plasma glucose (FPG), serum creatinine (SCR), fasting insulin, glycated hemoglobin A1c (HbA1c) and blood urea nitrogen (BUN) concentrations.

The AIP is assessed depending on TG and HDL-C concentrations as follows:


lg[TG(mmol/L)/HDL−C(mmol/L)]

### Traditional echocardiographic parameters

All obese patients underwent echocardiographic examinations performed by professional echocardiographers using an ultrasound tool (Vivid E9; GE Vingmed Ultrasound, Norway). During the investigation, an electrocardiogram was synchronously connected, and the heart rate (HR) was recorded.

The left atrial diameter (LAD), LV diameter (LVD), LV posterior wall thickness diameter (LVPWD) and interventricular septum thickness diameter (IVSD) were assessed at the end-diastole in the parasternal long-axis view (18). LV mass (LVM), LV hypertrophy (LVH) and relative wall thickness were obtained through calculations based on these parameters. The early (E) and late diastolic peak flow velocities (A) of the mitral valve were measured, and the E/A ratio was measured accordingly. Moreover, the early diastolic peak flow velocity (e′) of the lateral mitral annulus was assessed via the Doppler method, and the E/e′ ratio was subsequently obtained (19). M-mode echocardiography was utilized to measure the Mitral annular plane systolic excursion (MAPSE) (20). LV end-systolic volume (LVESV), LVEF, and LV end-diastolic volume (LVEDV) were calculated via the biplane Simpson’s technique. The left atrial volume (LAV) was obtained by taking the average value of the apical four-chamber view and apical two-chamber view. LVM index (LVMI), LAV index (LAVI), LVEDV index (LVEDVI), LVESV index (LVESVI) were obtained by correcting for differences among different obese patients based on BSA.

Left ventricular hypertrophy (LVH) was defined as an LVMI >115 g/m^2^ in men and >95 g/m^2^ in women, according to the recommendations of the American Society of Echocardiography and the European Association of Cardiovascular Imaging (18).

### STE

In the apical four-, two-, and three-chamber views, speckle-tracking technology was utilized to automatically identify LV endocardium and draw regions of interest for tracking LV myocardial motion (Software: EchoPAC, Version: 204, GE Vingmed Ultrasound, Norway). On this basis, the LV GLS was further analyzed and obtained. LV GLS is expressed as an absolute value; lower values indicate reduced myocardial longitudinal deformation and worse systolic function. LV GLS was measured independently from LVEF and LVH, which were derived from separate volumetric and dimensional measurements, respectively.

### Statistical analysis

After measuring and calculating AI*p* values of obese participants, obese participants were separated into 4 groups depending on quartiles.

Evaluate whether the parameters were normal distribution by Shapiro–Wilk test. One-way analysis of variance (ANOVA) was utilized to compare the differences in normally distributed data among the four groups, and the parameters are expressed as mean±SDs. To compare differences in abnormally distributed continuous variables among the 4 groups, the Kruskal-Wallis test was utilized, with the parameters presented as medians (interquartile ranges). To compare the categorical data among the 4 groups, the x^2^ test was employed, and the parameters are expressed as absolute values (percentages).

The relationship between potential risk factors (clinical data, biochemical parameters and echocardiographic indicators) and LV GLS was evaluated by correlation analysis.

To evaluate the independent correlation between absolute LV GLS < 20% (18) and AIP across four groups, 5 logistic regression models with forced-entry were constructed: the Crude model; Model 1, adjusted for age and sex; Model 2, further adjusted for BMI and HR; Model 3, further adjusted for LVH and LVEF; and Model 4, further adjusted for HT, diabetes mellitus, BUN, and SCR.

The sample size was evaluated based on both the events-per-variable (EPV) principle and the pmsampsize framework for multivariable logistic regression models. Assuming an anticipated C-statistic of 0.80, an outcome prevalence of 30%, and 13 candidate parameters, the minimum required sample size was estimated to be 448 participants with at least 135 outcome events. Since the final study population exceeded this threshold, the study was considered adequately powered for multivariable regression and incremental predictive analyses.

Base Model were LVH and LVEF. Traditional Model 1 were TG abnormality, LVH, and LVEF. Traditional Model 2 were HDL abnormality, LVH, and LVEF. The AIP model were AIP, LVH and LVEF. Receiver operating characteristic (ROC) curves were generated, and the area under the curve (AUC) was calculated to assess model discrimination. ROC curves were constructed using predicted probabilities derived from each logistic regression model, with LV GLS < 20% as the binary outcome. The y-axis represents sensitivity (true positive rate) and the x-axis represents 1-specificity (false positive rate). Differences between ROC curves were compared using the Delong test. Continuous net reclassification improvement (NRI) and integrated discrimination improvement (IDI) were additionally calculated to evaluate improvements in risk reclassification and discrimination after incorporation of AIP. Decision curve analysis (DCA) was performed to assess the clinical net benefit of different models across a range of threshold probabilities. The aim is evaluated whether AIP provided incremental value beyond traditional lipid abnormality indicators and conventional echocardiographic parameters.

All statistical analyses in this study were performed using R software (version 4.6.0, R Foundation for Statistical Computing, Vienna, Austria) and SPSS 23.0 (SPSS Inc., United States). *p* < 0.05 was considered statistically significant.

## Results

### Clinical data and biochemical parameters

A total of 1,070 individuals with obesity participated in this investigation before its initiation. Among them, 455 patients were omitted because of poor image quality and concurrent cardiovascular diseases, leaving 615 obese patients ultimately involved in the investigation. We classified the participants into 4 groups depending on the AIP quartiles: Group 1 (AIP ≤ 0.0081) (*n* = 153), Group 2 (0.0081 < AIP ≤ 0.1785) (*n* = 155), Group 3 (0.1785 < AIP ≤ 0.3661) (*n* = 154) and Group 4 (AIP > 0.3661) (*n* = 153). Table 1 illustrates the clinical and biochemical parameters of the four groups.

**Table 1:** Clinical characteristics of participants by quartiles of the atherogenic index of plasma.

Characteristic	Group1 (AIP ≤ 0.0081) *n* = 153	Group2 (0.0081 < AIP ≤ 0.1785) *n* = 155	Group3 (0.1785 < AIP ≤ 0.3661) *n* = 154	Group4 (AIP > 0.3661) *n* = 153	*p* value
Age, year	30.10 ± 7.31	31.40 ± 8.02	32.27 ± 7.99	32.07 ± 6.86	0.053
Male, *n* (%)	12(7.8)	29 (18.7) ^a^	45 (29.2) ^a^	70 (45.8) ^abc^	<0.001
Height, cm	164.51 ± 6.44	166.94 ± 7.91 ^a^	167.44 ± 8.68 ^a^	169.46 ± 8.08 ^abc^	<0.001
Weight, kg	100.32 ± 17.14	104.75 ± 19.59	108.39 ± 23.35 ^a^	107.65 ± 20.61 ^a^	0.002
BMI, kg/m^2^	37.00 ± 5.54	37.39 ± 5.11	38.36 ± 5.76	37.23 ± 4.87	0.122
BSA, m^2^	2.13 ± 0.24	2.21 ± 0.29 ^a^	2.26 ± 0.34 ^a^	2.26 ± 0.30 ^a^	0.001
Waist, cm	114.48 ± 13.08	117.00 ± 13.74	119.22 ± 14.65	116.12 ± 12.72	0.076
SBP, mmHg	129.91 ± 14.99	133.10 ± 15.30	132.66 ± 16.60	138.54 ± 19.85 ^abc^	<0.001
DBP, mmHg	84.78 ± 10.71	86.37 ± 11.90	87.24 ± 12.48	90.84 ± 13.08 ^abc^	<0.001
HR, bpm	81.11 ± 11.82	82.17 ± 13.58	82.62 ± 12.38	84.87 ± 13.80	0.076
FPG, mmol/L	5.39 (5.12,5.88)	5.70 (5.19,6.60) ^a^	5.73 (5.20,6.81) ^a^	6.45 (5.54,8.92) ^abc^	<0.001
Fasting insulin, pmol/ml	182.90 (124.20,256.35)	221.80 (151.20,343.75) ^a^	211.15 (154.23,319.58) ^a^	253.70 (159.28,378.08) ^a^	<0.001
HbA1c, %	5.60 (5.40,5.90)	5.80 (5.50,6.20) ^a^	5.90 (5.50,6.50) ^a^	6.30 (5.70,7.53) ^abc^	<0.001
TC, mmol/L	4.37 (3.99,4.93)	4.75 (4.24,5.33) ^a^	4.85 (4.29,5.39) ^a^	5.21 (4.46,5.99) ^abc^	<0.001
TG, mmol/L	0.98 (0.81,1.16)	1.43 (1.27,1.64) ^a^	1.90 (1.68,2.12) ^ab^	3.20 (2.61,4.13) ^abc^	<0.001
HDL-C, mmol/L	1.25 (1.12,1.42)	1.16 (1.04,1.29) ^a^	1.05 (0.97,1.13) ^ab^	0.95 (0.82,1.10) ^abc^	<0.001
LDL-C, mmol/L	2.82 (2.50,3.21)	3.23 (2.74,3.61) ^a^	3.25 (2.77,3.76) ^a^	3.34 (2.72,3.91) ^a^	<0.001
BUN, mmol/L	4.60 (3.90,5.60)	4.60 (3.80,5.30)	4.70 (3.68,5.80)	4.60 (3.70,5.30)	0.721
SCR, μmol/L	57.00 (51.00,63.00)	56.00 (49.00,64.00)	57.00 (50.00,68.00)	62.00 (51.00,71.00) ^ab^	0.005
AIP	−0.10 (−0.18, −0.04)	0.10 (0.05,0.14) ^a^	0.26 (0.23,0.30) ^ab^	0.50 (0.42,0.64) ^abc^	<0.001
Complications, %					
Hypertension	39 (25.49)	52 (33.55)	60 (38.96)	82 (53.59) ^ab^	<0.001
Diabetes mellitus	16 (10.46)	34 (21.94) ^a^	46 (29.87) ^a^	75 (49.02) ^abc^	<0.001
Dyslipidaemia	31 (20.26)	65 (41.94) ^a^	108 (70.13) ^ab^	153 (100.00) ^abc^	<0.001

BMI: body mass index, BSA: body surface area, SBP: systolic blood pressure, DBP: diastolic blood pressure. HR: heart rate, FPG: fasting plasma glucose, HbA1c: glycated hemoglobin A1c, TC: total cholesterol, TG: triglyceride, HDL-C: high-density lipoprotein cholesterol, LDL-C: low-density lipoprotein cholesterol, BUN: blood urea nitrogen, SCR: serum creatinine. ^a^*p* < 0.05 between the 1st quartile and 2nd quartile, 3rd quartile, 4th quartile. ^b^*p* < 0.05 between the 2nd quartile and 3rd quartile, 4th quartile. ^c^*p* < 0.05 between the 3rd quartile and 4th quartile.

No significant variations were shown in BMI, age, and HR among the four groups (*p* > 0.05). Compared to those in Group 1, the proportions of males in Group 2, 3 and 4 showed significant variation (*p* < 0.05). The proportion of males in Group 4 was significant different from that in Groups 2 and 3 (*p* < 0.05).

No significant variations in BUN were shown among the 4 groups (*p* > 0.05). In Group 2, FPG, TC, TG, LDL-C, fasting insulin, and HbA1c were significantly higher than those in Group 1, while HDL-C in Group 2 was significantly lessened than that in Group 1. In Group 3, FPG, TC, fasting insulin, HbA1c, TG, and LDL-C were significantly higher than those in Group 1. TG in Group 3 displayed a significant elevation compared to Group 2, while HDL-C in Group 3 depicted a significant reduction compared to Groups 1 and 2. In Group 4, TG, FPG, SCR, fasting insulin, HbA1c, TC, and LDL-C were significantly heightened compared to Group 1. Compared to Group 2, FPG, HbA1c, TC, TG, and SCR in Group 4 displayed a significant elevation. FPG, HbA1c, TC, and TG in Group 4 were significantly increased than in Group 3. In addition, HDL-C in Group 4 displayed a significant reduction compared to Groups 1, 2, and 3.

Comparing Groups 1 and 2, Group 2 had a substantially higher AIP, Group 3 had a significantly higher AIP than Groups 1 and 2, and Group 4 had a significantly higher AIP than Groups 1, 2, and 3.

The percentage of individuals with concurrent HT in Group 4 was significantly greater than in Groups 1 and 2. The percentage of individuals with concurrent diabetes mellitus in Groups 2 and 3 was significantly increased than that in Group 1, and Group 4 had a significantly greater percentage of individuals with concurrent diabetes mellitus than Groups 1, 2, and 3. The proportion of individuals with concurrent dyslipidaemia in Group 2 was significantly increased than that in Group 1. The percentage of individuals with concurrent dyslipidaemia in Group 3 was significantly higher than that in Groups 1 and 2. In Group 4, the percentage of individuals with concurrent dyslipidaemia was significantly greater than in Groups 1, 2, and 3.

### Traditional and speckle-tracking echocardiographic parameters

The results of traditional and STE parameters are shown in Table 2. In Group 2, the absolute values of LVEF and LVGLS showed a significant decrease compared to Group 1. In Group 3, IVSD, LVPWD, LVD, and LAD were significantly increased than those in Group 1. The LVEF in Group 3 displayed a significant elevation compared to Group 2. In Group 4, IVSD, LVPWD, LVD, LVMI, LAD, and E/e′ were significantly greater than in Group 1. The LVEF and LVGLS absolute values in Group 4 displayed a significant reduction compared to Group 1. Compared to Group 2, IVSD, LVPWD, LVD, and LVMI in Group 4 displayed a significant elevation. Compared to Group 3, LVD, LVMI, LVESVI, and E/e′ in Group 4 displayed a significant elevation, while the LVEF and LVGLS absolute values in Group 4 were significantly reduced. The proportion of absolute LV GLS < 20% in Group 4 showed a significant elevation compared to Groups 1 and 3.

**Table 2:** Clinical characteristics of LA and LV functions stratified by quartiles of the atherogenic index of plasma.

Characteristic	Group1 (AIP ≤ 0.0081) *n* = 153	Group2 (0.0081 < AIP ≤ 0.1785) *n* = 155	Group3 (0.1785 < AIP ≤ 0.3661) *n* = 154	Group4 (AIP > 0.3661) *n* = 153	*p* value
Septal thickness, mm	9.71 ± 0.89	9.84 ± 1.13	10.04 ± 1.04 ^a^	10.26 ± 1.38 ^ab^	<0.001
Posterior wall thickness, mm	9.54 ± 0.98	9.73 ± 0.89	9.81 ± 1.00 ^a^	10.03 ± 1.38 ^ab^	0.001
LV diameter, mm	48.09 ± 3.43	48.67 ± 4.07	49.04 ± 4.47 ^a^	50.07 ± 4.66 ^abc^	<0.001
Relative wall thickness	0.40 ± 0.04	0.40 ± 0.04	0.40 ± 0.04	0.40 ± 0.05	0.794
LV mass, g	163.53 ± 36.04	170.68 ± 37.38	177.05 ± 47.92 ^a^	190.00 ± 65.45 ^abc^	<0.001
LV mass index, g/m^2^	76.80 ± 14.58	77.41 ± 13.75	78.36 ± 15.03	83.81 ± 23.99 ^abc^	0.001
LVH, %	12 (7.84)	11 (7.10)	9 (5.84)	11 (7.19)	0.920
LVEDV, ml	82.00 (71.50,96.00)	85.00 (70.00,100.00)	85.00 (72.00,101.00)	92.00 (77.50,104.50) ^a^	0.018
LVEDV index, ml/m^2^	39.69 (33.63,45.89)	39.52 (33.34,45.39)	38.62 (34.31,44.18)	40.99 (35.21,47.13)	0.165
LVESV, ml	30.00 (26.00,36.50)	32.00 (26.00,40.00)	31.00 (26.00,39.00)	35.00 (28.00,41.00) ^ac^	0.001
LVESV index, ml/m^2^	14.51 (12.29,16.86)	15.09 (12.28,17.81)	14.35 (12.04,16.86)	15.65 (13.27,18.23) ^c^	0.012
LVEF, %	62.78 ± 3.90	61.79 ± 3.78 ^a^	62.70 ± 4.03 ^b^	61.20 ± 4.13 ^ac^	0.001
MAPSE, mm	15.04 ± 1.84	14.82 ± 1.72	15.16 ± 1.71	14.67 ± 1.69	0.067
LA diameter, mm	36.50 ± 3.68	37.30 ± 3.40	37.55 ± 3.85 ^a^	38.14 ± 4.18 ^a^	0.002
LAV, ml	56.44 ± 13.49	55.30 ± 15.43	57.35 ± 15.23	56.92 ± 16.38	0.667
LAVI, ml/m^2^	27.45 ± 6.27	26.23 ± 7.11	26.73 ± 6.44	26.25 ± 6.59	0.326
E, m/s	0.82 ± 0.15	0.82 ± 0.16	0.79 ± 0.15	0.79 ± 0.16	0.112
A, m/s	0.69 ± 0.15	0.71 ± 0.17	0.70 ± 0.16	0.72 ± 0.19	0.678
E/A	1.23 ± 0.31	1.21 ± 0.33	1.18 ± 0.33	1.20 ± 0.61	0.734
e′, m/s	0.12 ± 0.02	0.11 ± 0.02	0.11 ± 0.02	0.11 ± 0.06	0.436
E/e′	7.27 ± 1.76	7.53 ± 1.53	7.33 ± 1.76	7.89 ± 2.35 ^ac^	0.018
LV GLS, %	−20.02 ± 2.55	−19.3 ± 2.42 ^a^	−19.54 ± 2.94	−18.29 ± 3.13 ^abc^	<0.001
LV GLS < -20%, %	68 (44.44)	89 (57.42)	78 (50.65)	100(65.36) ^ac^	0.002

LV, Left ventricular; LAV, Left atrial volume; LVH, Left ventricular hypertrophy; LVEDV, Left ventricular end-diastolic volume; LVESV, Left ventricular end-systolic volume; LVEF, Left ventricular ejection fraction; MAPSE, Mitral annular plane systolic excursion; E, Peak velocity during early diastole of the anterior mitral leaflet; A, peak velocity during late diastole of the anterior mitral leaflet; e’, peak early diastolic annular velocities using TDI by averaging the values at the septum and lateral positions. GLS, global longitudinal strain. ^a^*p* < 0.05 between the 1st quartile and 2nd quartile, 3rd quartile, 4th quartile. ^b^*p* < 0.05 between the 2nd quartile and 3rd quartile, 4th quartile. ^c^*p* < 0.05 between the 3rd quartile and 4th quartile.

### Correlation analysis

Table 3 illustrates the outcomes of correlation analysis. Sex, HDL-C, and LVEF were significantly negatively correlated with LVGLS in obese patients. HbA1c, BMI, SBP, SCR, FPG, HR, TG, BUN, AIP, and LVH were significantly positively linked to LVGLS in obese patients.

**Table 3:** Correlation tests of potential risk factors for LV GLS.

Parameters	LV GLS	
	*r*	*p* value
Age, years	−0.072	0.073
Sex, male	−0.389	<0.001
HR, bpm	0.218	<0.001
BMI, kg/m^2^	0.395	<0.001
SBP, mmHg	0.317	<0.001
FPG, mmol/L	0.125	0.002
HbA1c, %	0.187	<0.001
TC, mmol/L	0.044	0.277
TG, mmol/L	0.182	<0.001
HDL-C, mmol/L	−0.188	<0.001
LDL-C, mmol/L	0.0311	0.439
BUN, mmol/L	0.136	0.001
SCR, μmol/L	0.180	<0.001
AIP	0.244	<0.001
LVH	0.181	<0.001
LVEF	−0.529	<0.001

### Multivariate regression model

Parameters with *p* < 0.05 in the correlation analysis were regarded as adjustment variables for establishing the multivariate regression model. In addition, parameters that might be confounding factors based on literature and clinical judgment were also considered as adjustment variables for building the multivariate regression model. Table 4 illustrates the results of the establishment of the multivariate regression model.

**Table 4:** Multivariable logistic regression analysis of absolute LV GLS < 20% by AIP quartiles.

Groups	Crude model		Model 1		Model 2		Model 3		Model 4	
	OR (95%CI)	*p* value	OR (95%CI)	*p* value	OR (95%CI)	*p* value	OR (95%CI)	*p* value	OR (95%CI)	*p* value
Group1(AIP ≤ 0.0081)	Reference	—	Reference	—	Reference	—	Reference	—	Reference	—
Group2(0.0081 < AIP ≤ 0.1785)	11.097 (2.134–57.723)	0.004	7.122 (1.295–39.168)	0.024	5.929 (1.007–34.893)	0.049	5.478 (0.825–36.388)	0.078	4.054 (0.572–28.755)	0.161
Group3(0.1785 < AIP ≤ 0.3661)	2.049 (0.713–5.889)	0.183	1.541 (0.495–4.801)	0.456	1.135 (0.346–3.725)	0.834	1.052 (0.289–3.837)	0.938	0.884 (0.229–3.421)	0.859
Group4(AIP > 0.3661)	5.104 (2.669–9.760)	<0.001	2.869 (1.372–5.998)	0.005	3.176 (1.443–6.992)	0.004	3.120 (1.300–7.486)	0.011	2.612 (1.007–6.778)	0.048

Model 1: adjusted for age and sex. Model 2: adjusted for model 2 covariates + BMI and HR. Model 3: adjusted for model 3 covariates + LVH and LVEF. Model 4: adjusted for model 4 covariates + hypertension, diabetes mellitus, BUN and SCR. OR, Odds ratio; 95% CI: 95% confidence interval. An OR >1.0 indicates higher odds of absolute LV GLS <20% compared with the reference group (Group 1).

Group 1 was considered as the reference group. No factors were adjusted in the crude model. Group 2 and Group 4 were significantly related to absolute LV GLS < 20%. After the adjustment of Model 1 for age and gender, Group 2 and Group 4 were significantly related to absolute LV GLS < 20%. After the adjustment of Model 2 for BMI and HR based on Model 1, Group 2 and Group 4 were significantly related to absolute LV GLS < 20%. After adjustment for LVH and LVEF in Model 3 based on Model 2, Group 4 was significantly linked to absolute LV GLS < 20%. After adjusting for HT, diabetes mellitus, BUN and SCR. in Model 4 based on Model 3, Group 4 remained significantly associated with absolute LV GLS < 20%.

### Incremental value of AIP beyond conventional echocardiographic and lipid parameters

**Figure 2:**
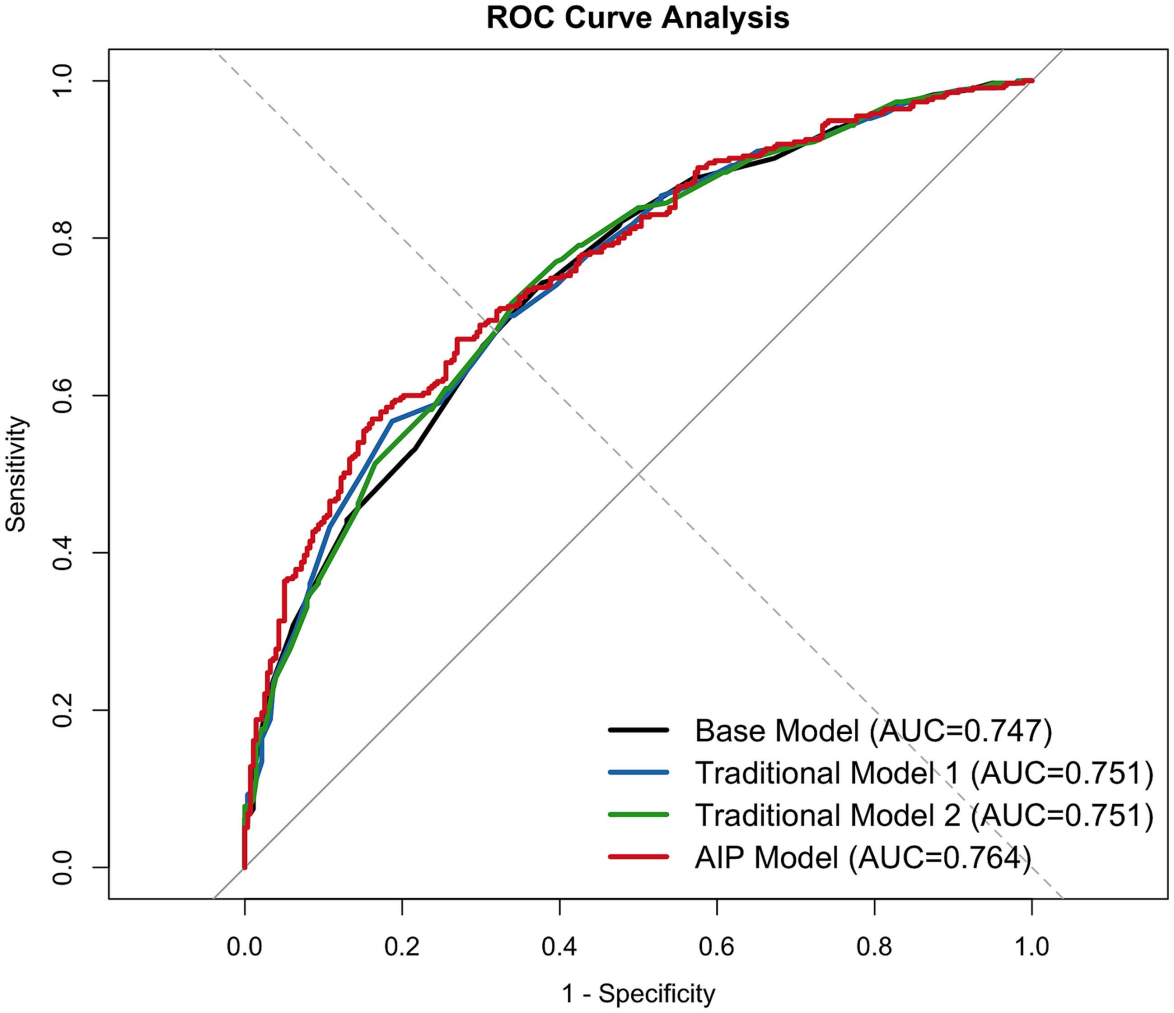
ROC curves comparing the discriminative performance of four models for identifying subclinical LV systolic dysfunction (absolute LV GLS < 20%). The y-axis represents sensitivity (true positive rate) and the x-axis represents 1-specificity (false positive rate); a higher AUC indicates better discrimination. AUC, area under the curve; AIP, atherogenic index of plasma; LVH, left ventricular hypertrophy; LVEF, left ventricular ejection fraction; TG, triglyceride; HDL-C, high-density lipoprotein cholesterol.

ROC analysis demonstrated that the AIP model exhibited the highest discriminative performance (AUC = 0.764, 95% CI: 0.727–0.801), compared with the Base Model (AUC = 0.747,95% CI: 0.709–0.785), Traditional Model 1 (AUC = 0.751, 95% CI: 0.713–0.789) and Traditional Model 2 (AUC = 0.751, 95% CI: 0.713–0.789). DeLong testing demonstrated that Traditional Model 1 and Traditional Model 2 did not significantly improve discrimination beyond the Base Model (*p* = 0.311 and *p* = 0.321, respectively). In contrast, AIP Model significantly improved model discrimination compared with the Base Model (*p* = 0.019) (Table 5 and Figure 2). The full regression coefficients, standard errors, and intercept of the AIP model are presented in Supplementary Table S1.

**Table 5:** Comparison of predictive performance among different models.

Model	AUC (95% CI)	Delong *p* value^*^	NRI^†^	IDI^†^
Base Model (LVH + LVEF)	0.747 (0.709–0.785)	Reference	Reference	Reference
Traditional Model 1 (TG abnormality + LVH + LVEF)	0.751 (0.713–0.789)	0.311	0.120	0.004
Traditional Model 2 (HDL-C abnormality + LVH + LVEF)	0.751 (0.713–0.789)	0.321	0.117	0.005
AIP Model (AIP + LVH + LVEF)	0.764 (0.727–0.801)	0.019	0.128	0.020

*DeLong test compared with the Base Model (LVH + LVEF). ^†^NRI and IDI were calculated relative to the base model.

AIP model achieved the highest improvement in risk reclassification and discrimination, with a continuous NRI of 0.128 and an IDI of 0.020 relative to the Base Model. By comparison, Traditional Model 1 resulted in a continuous NRI of 0.120 and an IDI of 0.004, whereas Traditional Model 2 yielded a continuous NRI of 0.117 and an IDI of 0.005 (Table 5).

**Figure 3:**
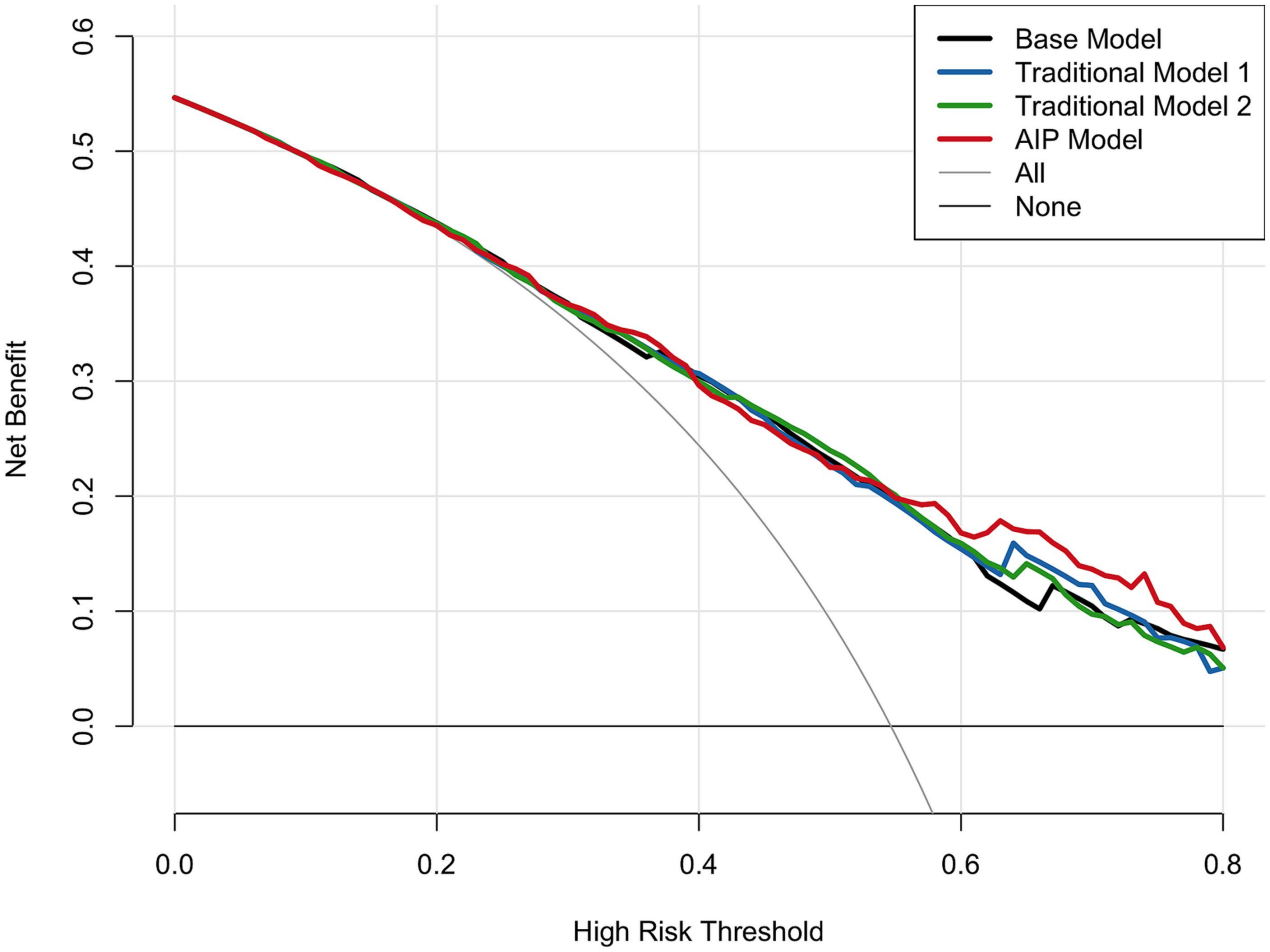
Decision curve analysis comparing the net clinical benefit of Base Model, Traditional Model 1, Traditional Model 2, and the AIP model across a range of threshold probabilities. The y-axis represents net benefit (true positives minus weighted false positives per patient); the x-axis represents threshold probability (the minimum probability of subclinical LV SD at which a clinician would act). A model with a higher net benefit across clinically relevant threshold probabilities is preferred. The grey line represents treat-all; the horizontal line at zero represents treat-none.

DCA showed broadly comparable clinical utility among the evaluated models across most threshold probabilities (Figure 3). However, the AIP model tended to yield slightly higher net benefit than the Base Model and traditional lipid models at intermediate-to-high threshold probabilities, suggesting a modest incremental clinical value of AIP for identifying subclinical LV SD.

## Discussion

The present study found that: (1) LV GLS in Group 4 (AIP > 0.3661) was significantly impaired compared with that in Groups 1 (AIP ≤ 0.0081), 2 (0.0081 < AIP ≤ 0.1785) and 3 (0.1785 < AIP ≤ 0.3661). (2) After adjustment for potential risk factors, AIP > 0.3661 was an independent influencing factor for absolute LV GLS < 20%.

Obesity is a main global health concern, with its occurrence heightening annually in developed and developing countries (21, 22). Many investigations have illustrated that obesity is significantly related to the incidence and progression of cardiovascular disorders. Moreover, the interrelationship and interaction between obesity and HT, diabetes mellitus, dyslipidaemia, and sleep apnoea syndrome can elevate the risk of cardiovascular complications among obese patients (23). Obesity is strictly linked to the incidence of coronary artery disease (CAD). In a large multinational cohort, each 5 kg/m^2^ increase in BMI was associated with a 25% higher prevalence of CAD and a 13% higher prevalence of obstructive CAD (24). Excessively stored adipose tissue in obese patients promotes the release of adipocytokines and induces insulin resistance and inflammatory responses, which contributes to the occurrence and progression of atherosclerosis (25). Obesity is closely linked to heart failure (HF). The risk of developing HF increases by 5% in males and 7% in females for each 1 kg/m^2^ elevation in BMI (26). Studies on HF have shown that 32–49% of HF patients have concurrent obesity, and 31–40% have overweight concurrently (23). Currently, the impairment of cardiac function caused by changes in cardiac structure and function observed only among obese patients is referred to as obese cardiomyopathy (27). Studies have indicated that this impairment is related to multiple mechanisms, including abnormal stimulation of the renin-angiotensin-aldosterone system, abnormal increase in inflammatory cytokines, and toxic effects of fat deposition on cardiomyocytes (27–29). Therefore, early assessment of cardiac function among obese patients is necessary. STE can be used to assess systolic function among cardiovascular disease patients, especially those in the subclinical stage with preserved LVEF (30). Previous studies have shown that LV GLS obtained by STE analysis can used to evaluate subclinical LV SD effectively in cardiovascular disorders including coronary heart disease and cardiomyopathy (31, 32). This parameter has also been used to assess LV SD effectively among obese patients (8, 10).

AIP is a novel parameter that can accurately predict cardiovascular diseases and their adverse prognosis. Ran et al. (33) illustrated that AIP was significantly related to the risk of adverse cardiovascular conditions in individuals with premature coronary artery disease and was an independent prognostic parameter for such patients. Wang et al. (34) found in their study on acute myocardial infarction (AMI) that AIP was significantly linked to 28-day in-hospital death in AMI patients, and an increase in AIP indicated a significant elevation in the death risk. Another study on cardiovascular-kidney-metabolic (CKM), it was found that elevated AIP was related to an elevated death risk in CKM patients and was significantly related to late-stage all-cause mortality, providing more new reference indicators for assessing the death risk in CKM patients (35). Nevertheless, no investigation has assessed the relationship between AIP and subclinical LV SD among obese patients. Therefore, this study innovatively adopted the AIP indicator and performed grouping analysis based on its quartiles to explore its correlation with LV systolic function among obese patients. There were significant differences in LVEF among the four groups. The LVEF in Group 4 was significantly decreased than that in Group 1 and Group 3. Although LVEF was decreased, all values remained within the normal range. In contrast, LV GLS was impaired to varying degrees in all groups, and Group 4 exhibited significantly LV GLS impairment than the other three groups. In the establishment of the multivariate regression model, after the adjustment for potential risk factors that may affect LV systolic function in obese patients, AIP > 0.3661 was an independent influencing factor for absolute LV GLS < 20%. An interesting finding of the present study was the non-monotonic association between AIP quartiles and impaired LV GLS. Group 2 showed a significantly higher risk of absolute LV GLS < 20% in the crude and partially adjusted models. However, this association became non-significant after further adjustment for LVH and LVEF. This attenuation suggests that the relationship observed in individuals with mildly elevated AIP may be largely mediated through early structural and functional cardiac remodeling. Once these intermediate cardiac parameters were considered, the independent contribution of moderate AIP elevation was substantially reduced. In contrast, Group 3 did not demonstrate a significant association with impaired LV GLS in any regression model. Although the exact mechanism remains uncertain, this finding may reflect biological heterogeneity within the intermediate AIP range and indicates that moderate elevations in AIP are insufficient to consistently influence myocardial deformation independent of other cardiometabolic factors. Notably, only participants in the highest AIP quartile (>0.3661) maintained a significant association with impaired LV GLS across all adjustment models. This pattern suggests the presence of a threshold effect rather than a simple linear dose–response relationship. Excessively elevated AIP levels may reflect a more severe atherogenic and insulin-resistant metabolic phenotype characterized by hypertriglyceridemia, reduced HDL-C levels, chronic inflammation, and endothelial dysfunction, all of which may directly contribute to subclinical LV SD. This provides an innovative parameter and additional reference value for the early assessment of subclinical LV SD among individuals with obesity.

Importantly, our findings extend beyond conventional association analysis. ROC analysis demonstrated that the AIP model achieved the highest discriminative ability among all evaluated models, while Delong testing confirmed significantly improved identified performance compared with the Base Model. Furthermore, NRI and IDI analyses indicated that incorporation of AIP improved both risk stratification and model discrimination, suggesting that AIP may better identify obese individuals at higher risk of subclinical LV SD. DCA further supported the clinical relevance of these findings. Although all evaluated models provided net clinical benefit across clinically relevant threshold probabilities, the AIP model demonstrated modestly greater net benefit than the Base Model and traditional lipid models, particularly at intermediate-to-high threshold probabilities. Collectively, these results indicate that AIP may serve as a practical and clinically useful marker for cardiovascular risk stratification in obese populations.

This finding suggests that in clinical practice, regular detection of AIP levels can be used to assess whether obese patients are likely to develop subclinical LV SD, with further examinations if necessary. When obese patients present with subclinical LV SD, BMI reduction through clinical interventions including gut microbiota regulation with dietary herbs (36), hormonal therapy and micronutrient supplementation (37) may reverse this subclinical cardiac dysfunction, halt further functional deterioration, and consequently decrease the risk of adverse cardiovascular events.

Several limitations of the present study should be acknowledged. Firstly, all participants were enrolled from a single medical center, and a considerable number of subjects were excluded due to comorbidities or poor image quality, which may cause potential selection bias. Accordingly, the clinical generalizability of our conclusions is limited. In the future, we will recruit participants from different regions and conduct multi-center, large-sample studies to enhance the validity and popularization value of the research findings. Secondly, data on smoking history, alcohol intake, physical activity and dietary habits were not collected in this study, which may introduce potential confounding factors. We will incorporate these indicators into further studies to deeply explore the mechanisms underlying subclinical LV SD in obese populations. Thirdly, this investigation did not assess the prognostic outcomes of obese patients. In future studies, our team will follow the obese population to assess whether myocardial function is reversed among obese patients with subclinical LV SD after drug or surgical intervention and whether such reversal is reflected by AIP. Furthermore, the AIP threshold of 0.3661 was derived from the quartile distribution of the present study population and has not been externally validated, its application to other populations should be approached with caution until confirmed in independent prospective cohorts.

## Conclusion

A high AIP index (>0.3661) is an independent influencing factor for subclinical LV SD among individuals with obesity.

## Data Availability

The raw data supporting the conclusions of this article will be made available by the authors, without undue reservation.
