# Supplementary material for: The atherogenic index of plasma is associated with subclinical cardiac systolic dysfunction among obese patients: a cross-sectional study
Source: Front Nutr. 2026 Jun 29;13:1845459. doi: 10.3389/fnut.2026.1845459 (PMC13357988; doi:10.3389/fnut.2026.1845459)
Supplement: Supplementary file 1 [file Table_1.DOCX]

Supplementary Table S1. Logistic regression coefficients for the AIP prediction model.

| Variable | β coefficient | Standard Error | Odds Ratio  (95% CI) | P value |
| --- | --- | --- | --- | --- |
| Intercept | 15.359 | 1.675 | — | <0.001 |
| AIP | 1.252 | 0.340 | 3.497  (1.797–6.804) | <0.001 |
| LVH | 0.741 | 0.385 | 2.097  (0.986–4.459) | 0.054 |
| LVEF | −0.249 | 0.027 | 0.780  (0.740–0.822) | <0.001 |

AIP: atherogenic index of plasma, LVH: left ventricular hypertrophy, LVEF: left ventricular ejection fraction.

Prediction equation: logit (P) = 15.359 + 1.252 × AIP + 0.741 × LVH − 0.249 × LVEF
